# Endogenous nitric oxide accumulation is involved in the antifungal activity of Shikonin against *Candida albicans*

**DOI:** 10.1038/emi.2016.87

**Published:** 2016-08-17

**Authors:** Zebin Liao, Yu Yan, Huaihuai Dong, Zhenyu Zhu, Yuanying Jiang, Yingying Cao

**Affiliations:** 1 School of Pharmacy, Second Military Medical University, Shanghai 200433, China

**Keywords:** *Candida albicans*, *CTA4*, nitric oxide, Shikonin, *YHB1*

## Abstract

The aim of the present study was to investigate the role of nitric oxide (NO) in the antifungal activity of Shikonin (SK) against *Candida albicans* (*C. albicans*) and to clarify the underlying mechanism. The results showed that the NO donors *S*-nitrosoglutathione (GSNO) and L-arginine could enhance the antifungal activity of SK, whereas the NO production inhibitor Nω-nitro-L-arginine methyl ester (L-NAME) attenuated antifungal action. Using the fluorescent dye 3-amino,4-aminomethyl-2′, 7-difluorescein, diacetate (DAF-FM DA), we found that the accumulation of NO in *C. albicans* was increased markedly by SK in a time- and dose-dependent manner. In addition, the results of real-time reverse transcription-PCR (RT-PCR) demonstrated that the transcription level of *YHB1* in *C. albicans* was greatly increased upon incubation of SK. Consistently, the *YHB1-*null mutant (*yhb1Δ/Δ*) exhibited a higher susceptibility to SK than wild-type cells. In addition, although the transcription level of *CTA4* in *C. albicans* was not significantly changed when exposed to SK, the *CTA4-*null mutant (*cta4Δ/Δ*) was more susceptible to SK. Collectively, SK is the agent found to execute its antifungal activity directly via endogenous NO accumulation, and NO*-*mediated damage is related to the suppression of *YHB1* and the function of *CTA4.*

## INTRODUCTION

*Candida albicans* (*C. albicans*) is the most important cause of fungal infection in humans, especially in immunocompromised patients.^1^ Available antifungals predominantly include azoles, echinocandins, polyenes and allylamines.^2^ Each of these classes of antifungals has its own distinct mode of action. Azoles target cytochrome *P*450 lanosterol 14α-demethylase, thereby impeding conversion of lanosterol to fecosterol and subsequently blocking ergosterol biosynthesis.^3^ Echinocandins interfere with cell wall synthesis by inhibiting β-1,-3-glucan synthase.^4^ Polyenes have an affinity to bind membrane sterols that results in the formation of aqueous pores, leading to leakage of crucial cellular components and subsequent cell death.^5^ Allylamines specifically target squalene epoxidase.^6^ However, the emergence of resistant strains of *Candida* because of prolonged use of drugs and that some pathogenic *Candida* species are intrinsically resistant to antifungals make candidiasis increasingly difficult to cure.^7^ The limited arsenal of antifungals constantly requires researchers to find new antifungals using novel mechanisms. In this context, herbal antifungals have acquired significance owing to their natural origin.^8^

Shikonin (SK), a molecule originally found in China, is the major constituent of the red pigment extracts from the roots of the plant *Lithospermum erythrorhizon Sieb. et Zucc* (*LE*). SK was reported to exhibit certain functions and was widely used to prepare an ointment to treat wounds, burns and hemorrhoids in Japan;^9^ to induce necrosis or apoptosis through generating reactive oxygen species to treat gastric cancers;^10^ and to serve possible therapeutic roles in brain disorders involving uncontrolled inflammatory responses.^11^ We recently found that SK exerted an antifungal effect on almost all *C. albicans* isolates tested. More importantly, the action of SK was shown to be >16 times higher than that of fluconazole against some fluconazole-resistant *C. albicans* isolates. However, although mitochondrial aerobic respiration and endogenous reactive oxygen species were identified as being involved in the antifungal activity of SK,^12^ the intrinsic mechanisms of the antifungal activity of SK have not been fully determined.

Nitric oxide (NO), an essential messenger involved in numerous physiological functions, has attracted much attention for its antimicrobial and antifungal activity and its synergistic effects when co-delivered with chemotherapeutic drugs.^13, 14^ *S*-nitrosoglutathione (GSNO), a potent NO donor formed by the interaction of NO with intracellular glutathione, exhibited antimicrobial activity against methicillin-resistant *Staphylococcus aureus*, *Escherichia coli*, *Klebsiella pneumoniae* and *Pseudomonas aeruginosa*,^15^ and the NO precursor L-arginine was used against *Salmonella*.^16^ In addition, the significant increase of fecal oocyst shedding after treatment with the NO synthase inhibitor Nω-nitro-L-arginine methyl ester (L-NAME) partly suggests that arginine-derived NO may reduce the parasite load in experimental cryptosporidiosis.^17^ Heilman *et al.*^18^ indicated that the hyphal form of the fungus was more susceptible to a biocompatible NO-donating material that delivers NO that is induced using light. However, the direct role of endogenous NO involved in the antifungal activity of antimycotics has not yet been studied. In this study, we demonstrated the endogenous accumulation of NO in *C. albicans* when treated with SK, and we clarified the intrinsic mechanisms. We used GSNO, L-arginine and L-NAME to show that the antifungal efficacy of SK was directly related to the intracellular levels of NO. Utilizing real-time reverse transcription-PCR (RT-PCR), the nitrosative stress was found to be mediated by *YHB1*, the only recognized gene that has been reported to encode the key NO-detoxifying enzyme, flavohemoglobin/nitric oxide dioxygenase,^19^ as well as *CTA4*, the first reported regulator of *YHB1* in *C. albicans*.^20^

## MATERIALS AND METHODS

### Strains, media and compounds

All strains used in this study are listed in Table 1. Strains were routinely maintained on Sabouraud dextrose agar and propagated in liquid yeast extract peptone dextrose (YPD) medium (1% w/v yeast extract, 2% w/v peptone and 2% w/v dextrose), peptone, yeast extract and agar were supplied by Becton Dickinson & Co. (Sparks, MD, USA). Batches of media were inoculated from YPD agar plates containing freshly grown *C. albicans* and were incubated overnight in an orbital shaker at 30 °C. Before being used in the following experiments, blastospores were harvested and washed twice in sterile phosphate-buffered saline (PBS; 10 mM phosphate buffer, 2.7 mM potassium chloride and 137 mM sodium chloride (pH 7.4)). The cells were then suspended in a yeast nitrogen base (YNB; Difco, Becton Dickinson & Co.) medium supplemented with 50 mM glucose, counted in a hemocytometer and adjusted to the desired cell density. During the experiments, 6.4 mg/mL SK (National Institutes for Food and Drug Control, Peking, China) and 8 mg/mL GSNO (Sigma-Aldrich, St Louis, MO, USA) in dimethyl sulfoxide, 10 mg/mL L-arginine and 100 mg/mL L-NAME (Sigma-Aldrich) in ultrapure distilled water and 5 mM NO fluorescent probe (3-Amino,4-aminomethyl-2', 7-difluorescein, diacetate (DAF-FM DA); Beyotime Institute Biotechnology, Jiangsu, China) were used as stock solutions and added to the culture suspensions to obtain the required concentrations.

### Survival rate test

The antifungal activities of SK in the absence or presence of GSNO, L-NAME or L-arginine were assessed as described previously.^23^ Briefly, after incubation for 1 h, samples were washed, resuspended in PBS and placed on YPD agar plates. The fraction of viable cells was determined by counting the colonies and by calculating the percentage of surviving *C. albicans* cells relative to the control treatment.

### Endogenous nitric oxide measurement

Intracellular levels of NO and the accumulation of NO in *C. albicans* were measured with DAF-FM DA. Briefly, culture suspensions were treated with the compounds; at different times, the samples were taken and washed with PBS, and then DAF-FM DA was added to the final concentration of 5 μM. The samples were incubated for 30 min in the dark at 30 °C, and then washed three times with PBS. To detect intracellular levels of NO, 300 μL of cell suspension was analyzed using a flow cytometer with excitation at 495 nm and emission at 515 nm set for DAF-FM DA, after which the samples were resuspended in 1 mL of PBS. To measure the accumulation of NO in *C. albicans*, the samples were resuspended in 500 μL of PBS, and then 10 μL of cell suspension was dropped on a clean glass sheet and analyzed using a Confocal Laser Scanning Microscope with excitation at 495 nm and emission at 515 nm for DAF-FM DA. The cover glass was added and then sealed with nail polish.

### Real-time reverse transcription-PCR

RNA isolation and real-time RT-PCR were performed as described previously,^24^ with some modifications. The isolated RNA was resuspended in diethyl pyrocarbonate-treated water. The OD260 and OD280 were measured. First-strand complementary DNAs (cDNAs) were synthesized from 3 μg of total RNA in a 60 μL reaction volume using the cDNA synthesis kit for RT-PCR (TaKaRa Biotechnology, Dalian, China). Independent quantitative real-time PCR was performed in triplicate using the Lightcycler system (Roche Diagnostics, GmbH, Mannheim, Germany). SYBR Green I (TaKaRa) was used to visualize and monitor the amplified product in real time according to the manufacturer's protocol. *YHB1* was amplified with the forward primer 5′-TGT TAG ACA TGT CCC AGG TGG-3' and the reverse primer 5'-CCA ATA CCA CCA GCA ACG AA-3'. The PCR protocol consisted of denaturation (95 °C for 10 s), 40 cycles of amplification and quantification (95 °C for 5 s, 60 °C for 34 s with a single fluorescence measurement), a melting curve analysis (60 °C–95 °C with a heating rate of 0.1 °C per s and a continuous fluorescence measurement) and, finally, cooling to 40 °C. A standard curve for each primer set was performed with 1:10, 1:25, 1:50, 1:100, 1:250 and 1:500 dilutions of the cDNAs. The slopes of the standard curves were within 10% of 100% efficiency. The change in fluorescence of SYBR Green I dye in every cycle was monitored using the Lightcycler system software, and the threshold cycle (*CT*) above background for each reaction was calculated. The *CT* value of 18S rRNA (amplified with the forward primer 5′-TCT TTC TTG ATT TTG TGG GTG G-3′ and the reverse primer 5′-TCG ATA GTC CCT CTA AGA AGT G-3′) was subtracted from that of the tested genes to obtain a *ΔC*_*T*_ value. The *ΔC*_*T*_ value of an arbitrary calibrator was subtracted from the *ΔC*_*T*_ value of each sample to obtain a *ΔΔC*_*T*_ value. The gene expression level relative to the calibrator was expressed as 2^*−ΔΔC*^_*T*_.

### Sensitivities of *C. albicans* mutants to Shikonin

The antifungal susceptibility test was performed using the Clinical and Laboratory Standards Institute (Document M27-A) method.^25^ Briefly, the initial concentrations of *CTA4-*null mutant (°C), *YHB1-*null mutant (*yhb1Δ/Δ*) and the wild type in RPMI-1640 medium were adjusted to 1 × 10^3^ colony-forming units (CFUs)/ml, whereas the final concentration of SK was 0.125–64 μg/mL. After the plates had been incubated at 30 °C for 24 h, the optical density of each well was measured at 600 nm (OD_600_). Each isolate was tested in triplicate, and the MIC_80_ values were determined as the lowest concentration of the agent that inhibited growth by 80% compared with that of the drug-free wells.^12^ To obtain a representative growth curve, strains in the desired cell density (1 × 10^5^ CFUs/mL) were treated with certain concentrations of SK in YPD liquid medium that were incubated overnight in an orbital shaker at 30 °C; the control group received only dimethyl sulfoxide. Meanwhile, a sample was taken to measure the OD_600_ value at different times.

### Statistical criteria

Data are expressed as the mean±SD of the independent assays in triplicate. Student's *t*-test was employed to assess the significance of the differences. A *P-*value of <0.05 or <0.01 was considered statistically significant.

## RESULTS

### The effect of the nitric oxide donor on the activity of Shikonin

**Figure 1:**
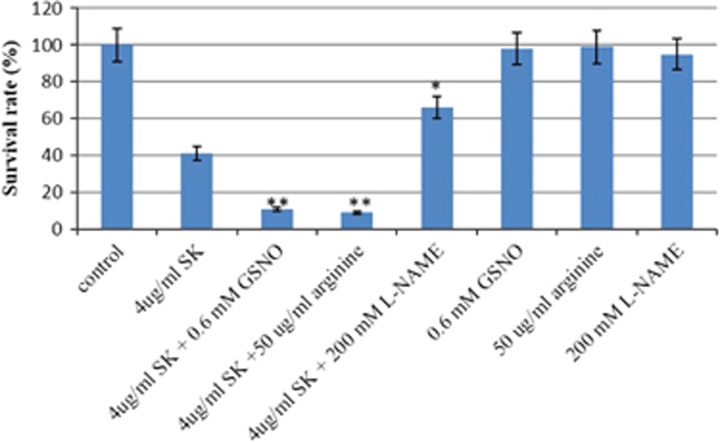
The effect of NO on the antifungal activity of SK against *C. albicans*. Suspensions of *C. albicans* SC5314 were exposed to 4 μg/mL SK, 50 μg/mL arginine, 200 mM L-NAME, 0.6 mM GSNO or a combination for 2 h. The samples were then washed, resuspended in PBS and plated on YPD agar plates. The fraction of viable cells was determined by counting the colonies and calculating the percentage of surviving *C. albicans* cells relative to the control treatment. Data are expressed as the mean±SD of triplicate assays. *P<0.05; **P<0.01 compared with values from the treatment of 4 μg/mL SK.

GSNO and L-arginine are both donors of NO in cells. We examined the effect of GSNO and L-arginine on the activity of SK against *C. albicans* SC5314. To this end, half the maximal inhibitory concentration of SK (IC_50_) was used. Treatment of *C. albicans* suspension with 4 μg/mL SK resulted in ~40% viable cells compared with the untreated group (Figure 1). We used this concentration of SK to further investigate the effect of GSNO and L-arginine on the activity of SK against *C. albicans* SC5314. As shown in Figure 1, the treatment of the combination of 4 μg/mL SK with 0.6 mM GSNO resulted in an ~11% viable rate of *C. albicans*, whereas an ~9% survival rate was obtained when treated with 4 μg/mL SK and 50 μg/mL L-arginine. Meanwhile, there was no significant effect of GSNO or L-arginine on the viabilities of *C. albicans* cells.

### The effect of nitric oxide scavenger on the activity of Shikonin

To further our investigation, we examined the effect of the NO scavenger L-NAME on the antifungal activity of SK. The result showed that when incubated with 200 mM L-NAME, there was no significant change in the survival rate of *C. albicans* cells, although the rate was a little lower than the untreated group. When treated with a combination of 4 μg/mL SK with 200 μg/mL L-NAME, the viable rate of *C. albicans* cells became ~66%, whereas 4 μg/mL SK treatment still resulted in ~40% viable cells (Figure 1).

### Shikonin induced the accumulation of nitric oxide

**Figure 2:**
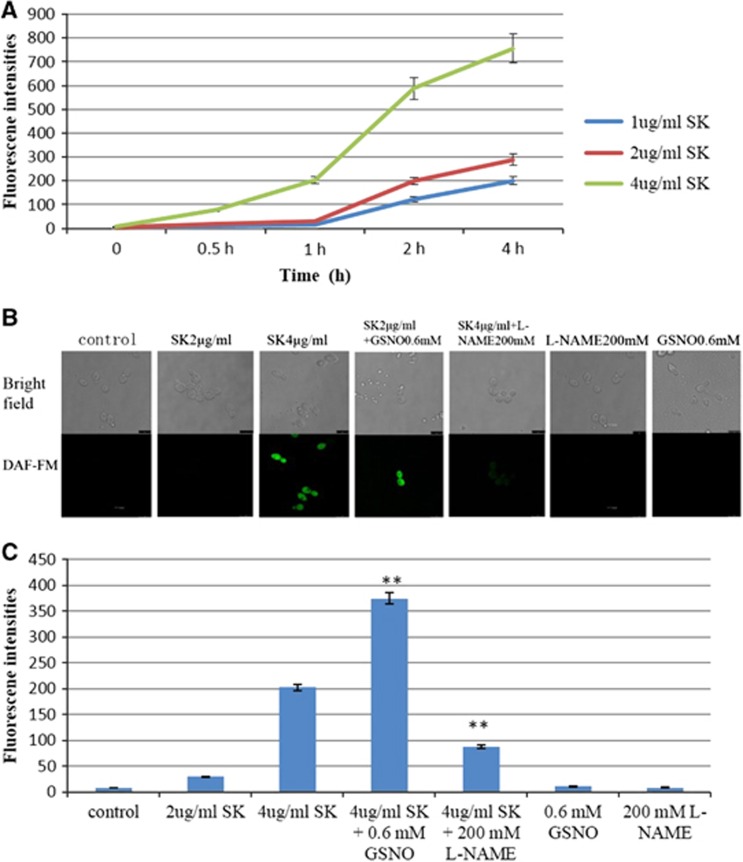
Measurement of intracellular levels of nitric oxide (NO) in *C. albicans* induced by SK. (**A**) *C. albicans* cells were treated with different concentrations of SK; at different times, samples were washed with PBS and analyzed for the production of intracellular NO using a flow cytometer with excitation at 495 nm and emission at 515 nm after incubation with a fluorescent probe. (**B**) Suspension cells were incubated with SK, GSNO, L-NAME or a combination for 2 h. NO accumulation was analyzed by the use of a Confocal Laser Scanning Microscope with excitation at 495 nm and emission at 515 nm in the green area. Bar: 7.5 μM. (**C**) *C. albicans* cells were treated with SK, GSNO, L-NAME or a combination for 2 h, then intracellular NO production was measured by a flow cytometer with excitation at 495 nm and emission at 515 nm after incubation with a fluorescent probe. Data are expressed as the mean±SD of triplicate assays. **P<0.01 compared with values from the 4 μg/mL SK treatment.

The survival rate indicated that the enhanced antifungal activity of SK might be related to NO. Thus, we detected the accumulation of endogenous NO in *C. albicans* cells using a confocal laser scanning microscope and flow cytometer. As is shown in our results, the incubation of SK resulted in an increased level of intracellular NO in a time- and dose-dependent manner (Figure 2A). Consistently, there was strong fluorescence in *C. albicans* cells when incubated with 4 μg/mL SK, and the fluorescence became weak when combined with 200 mM L-NAME. Conversely, there was no fluorescence in cells that were incubated with 2 μg/mL SK, but strong fluorescence was detected when combined with 0.6 mM GSNO. Meanwhile, cells treated with L-NAME and GSNO showed no NO accumulation (Figure 2B). Similar results were obtained when the intracellular levels of NO in *C. albicans* cells were measured using a flow cytometer (Figure 2C).

### Influence of Shikonin on the expression of *YHB1*

**Figure 3:**
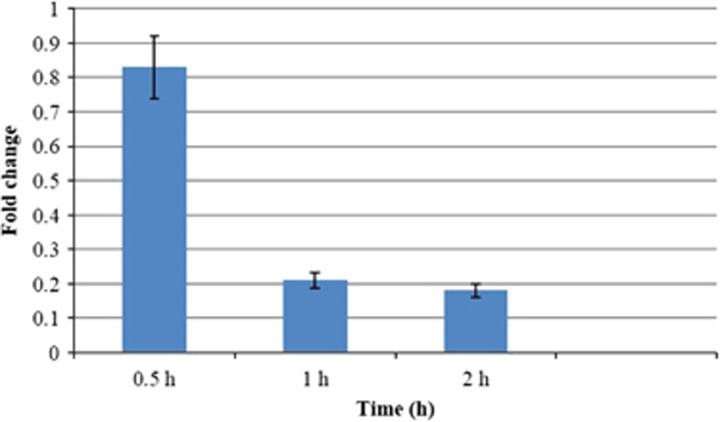
The effect of SK on the transcription of YHB1. *C. albicans* cells were treated with 2 μg/mL SK; a sample was taken at different times. Total RNA was extracted and reversely transcribed to cDNA. cDNA was then used for real-time quantitative PCR to detect expression levels of YHB1. Values represent the mean±SD of three replicates.

Because *YHB1* is the only recognized gene to encode the key NO-detoxifying enzyme flavohemoglobin/nitric oxide dioxygenase, we investigated the putative role of *YHB1* in the antifungal activity of SK against *C. albicans*. To this end, the effect of SK on the expression of *YHB1* in *C. albicans* cells was assessed. The results showed a marked change in the transcript levels of *YHB1* mRNA in a time-dependent manner; specifically, after exposure to 2 μg/mL SK for 0.5 h, there was no significant change in the transcript level. However, after 1 h of treatment, the *YHB1* transcript level had declined from 83% to 21%, and the greatest amount of reduction was obtained after *C. albicans* cells had been exposed to SK for 2 h, at which time the transcript level was 18%. Furthermore, *C. albicans* cells exhibited no significant change in the *YHB1* transcript level after being treated with 2 μg/mL SK for 4 h (Figure 3).

### The sensitivity of *YHB1* or *CTA4* mutant to Shikonin

**Figure 4:**
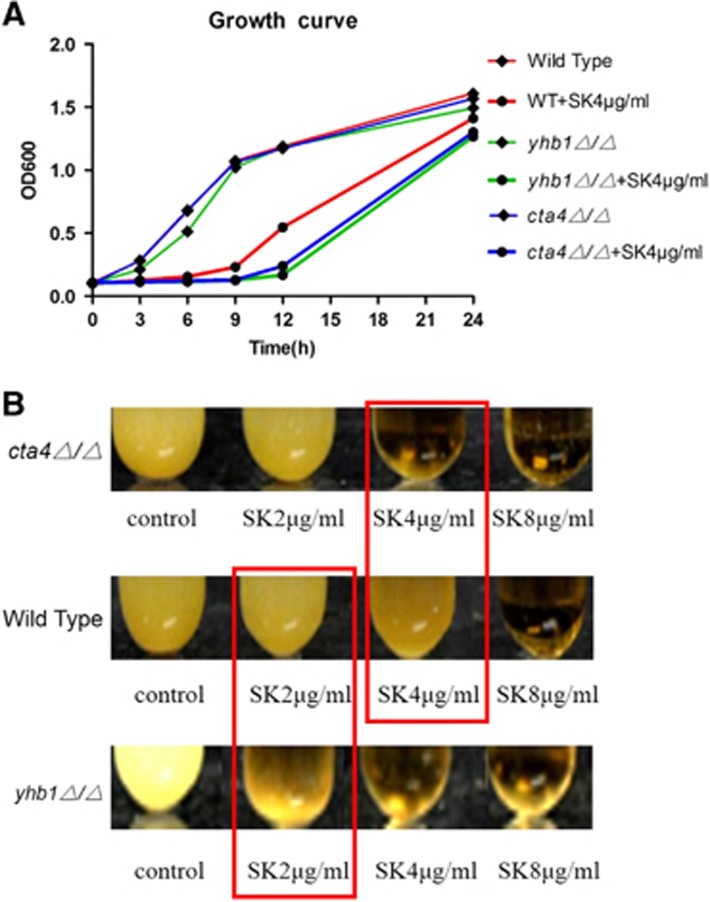
Sensitivity of *C. albicans* mutants to SK. Strains of the CTA4-null mutant (*cta4Δ/Δ*), YHB1-null mutant (*yhb1Δ/Δ*) and the wild type were treated with SK. All strains were incubated overnight in an orbital shaker at 30 °C. (**A**) At different times, a sample was taken to measure the OD600 value, from which a representative time-kill curve was obtained. (**B**) Photographs were taken after 24 h of incubation. Data are expressed as the mean±SD of triplicate assays.

The special role of *YHB1* in preventing *C. albicans* from NO-mediated damage implied that the antifungal activity of SK might be related to the suppression of *YHB1*. We examined the function of *YHB1* in the antifungal activity of SK against *C. albicans*, using the *YHB1-*null mutant (*yhb1Δ/Δ*). As shown in the results, the deletion of *YHB1* had no effect on the growth rate of *C. albicans* (Figure 4A), whereas the *YHB1-*null mutant *(yhb1Δ/Δ)* was more susceptible to SK than the wild type (Figure 4B and Table 2), and the growth rate of the *YHB1-*null mutant treated with 4 μg/mL SK was less than that of the wild type (Figure 4A).

Because *CTA4* regulates *YHB1* and Cta4p is the first protein reported to initiate a NO response in *C. albicans*, we investigated the role of *CTA4* in the antifungal activity of SK. The results showed that the deletion of *CTA4* increased the susceptibility of *C. albicans* to 4 μg/mL SK (Figure 4B and Table 2); the growth rate of the *CTA4-*null mutant (*cta4Δ/Δ*) was similar to that of the *YHB1-*null mutant (*yhb1Δ/Δ*) (Figure 4A), pointing to a clear relationship between the antifungal activity of SK and *CTA4*.

## DISCUSSION

The naphthoquinone pigment SK from the root of *L. erythrorhizon* has been reported to exhibit antimicrobial, anticancer, antithrombotic, anti-inflammatory and anti-atherosclerosis functions.^11^ Our previous work has indicated that SK could function as an antifungal compound against *C. albican*s by shifting mitochondrial aerobic respiration and inducing endogenous reactive oxygen species augmentation.^12^ In this study, the role of NO in the antifungal activity of SK against *C. albicans* was tested. The antifungal armamentarium for the treatment or prophylaxis of invasive fungal infections is very limited. In addition, the increased evolvement of *C. albicans* resistant to antifungal therapy requires a high treatment dose, increasing the risk of toxicity and side effects.^26^ There has been an urgent need to develop effective drugs with novel mechanisms for combating fungal diseases. NO is a gaseous radical that has diverse roles in biological systems, including vasodilation, signaling, defense and the destruction of microbes. NO release is part of the defense of higher organisms against invading microbial pathogens.^27^ NO has potent antifungal activity when produced by macrophages within the host,^13^ and a stable NO-releasing nanoparticle platform can serve as an innovative therapeutic to which the fungi are unlikely to evolve resistance.^28^ Our results showed that both GSNO, a store of NO,^15^ and L-arginine, a producer of NO by the catalytic action of constitutive and inducible NO synthase isoforms in cells,^16^ could enhance the antifungal activity of SK, whereas L-NAME, the competitive inhibitor of NO synthase,^17^ attenuated the action. These results prompted us to hypothesize that the enhanced antifungal activity of SK might also be due to increased nitrosative damage. As expected, the incubation of *C. albicans* cells with SK induced changes in the intracellular levels of NO in a time- and dose-dependent manner: the increased level of NO by SK was enhanced by GSNO, whereas L-NAME attenuated the action. David *et al.* have reported that fluconazole could increase the expression of genes related to nitrosative damage.^29^ However, the antifungal role of NO accumulation directly induced using antifungal agents has not been reported and, to our knowledge, this is the first report demonstrating that the accumulation of NO is directly involved in the antifungal activity of antimycotic compounds against fungi. As NO is produced by macrophages to kill *C. albicans* and fungi treated with a NO-releasing nanoparticle platform are unlikely to evolve resistance,^13, 28^ SK may serve as a potential agent to induce mortality of *C. albicans in vivo.*

*C. albicans* utilizes several mechanisms to counteract nitrosative stresses, including the active detoxification of NO via flavohemoglobins, scavenging NO via the antioxidant system and the upregulation of repair systems to counteract damage.^30^ Flavohemoglobins could metabolize NO to nitrate and protect microbes from NO-mediated damage, growth inhibition and killing by NO-releasing immune cells.^31^ In *C. albicans*, the most highly induced gene is *YHB1* that encodes the key NO-detoxifying enzyme flavohemoglobin/nitric oxide dioxygenase under nitrosative stresses.^19^ Our results showed that the transcription of *YHB1* dramatically declined in a time-dependent manner after the treatment of SK, and the *YHB1-*null mutant (*yhb1Δ/Δ*) was more susceptible to SK treatment than the wild-type cells.

How yeasts detect NO and which signaling pathways mediate NO responses remains unclear. Nevertheless, Chiranand *et al.*^20^ reported that *YHB1* expression was activated by the regulator *CTA4*, and the inactivation of *CTA4* inhibits *YHB1* induction in response to NO. Consistent with these findings, our investigation demonstrated that the *CTA4-*null mutant (*cta4Δ/Δ*) manifested a remarkable susceptibility to SK treatment, although the expression of *CTA4* exhibited no significant change in *C. albicans* when treated with SK (data not shown). This contradiction strongly suggests that *CTA4* may function in the post-translated level during the NO-mediated damage of SK, and SK may directly influence the proteins translated by *CTA4*, further regulating the function of *YHB1* to encode the key enzyme-detoxifying nitrosative damage. In addition, other regulators may exist that influence the transcription of *YHB1* in *C. albicans* to counteract nitrosative stresses. Sellam *et al.*^32^ have recently demonstrated that Cwt1p was a negative modulator of nitrosative stress resistance through direct transcriptional control of *YHB1*. Whether SK coordinates with the transcription factor Cwt1p that targets *YHB1* or affects other pathways requires further investigation.

In conclusion, SK is able to induce the accumulation of intracellular NO in *C. albicans*, which subsequently manifests nitrosative stresses, and the NO-mediated damage in *C. albicans* by SK is related to the suppression of *YHB1* and the function of *CTA4*. Our study sheds new light on the pathway in which *C. albicans* responds to nitrosative stresses.

## Figures and Tables

**Table 1:** *Candida albicans* strains used in this study

**Strain**	**Phenotype**	**Genotype**	**Reference**
SC5314		Prototroph	^21^
SN250	Reference strain Arg-	*his1Δ/his1Δ, leu2Δ::C. dubliniensis HIS1 /leu2Δ::C. maltosa LEU2, arg4Δ /arg4Δ, URA3/ura3Δ::imm434, IRO1/iro1Δ::imm434*	^22^
*cta4Δ/Δ*	orf19.7374 mutant Arg-	*his1Δ/his1Δ, leu2Δ /leu2Δ, arg4Δ /arg4Δ, URA3/ura3Δ::imm434, IRO1/iro1Δ::imm434, orf19.7374Δ::C. dubliniensisHIS1/orf19.7374Δ::C. maltosaLEU2*	^22^
*yhb1Δ/Δ*	orf19.3707 mutant Arg-	*his1Δ/his1Δ, leu2Δ /leu2Δ, arg4Δ /arg4Δ, URA3/ura3Δ::imm434, IRO1/iro1Δ::imm434, orf19.3707Δ::C. dubliniensisHIS1/orf19.3707Δ::C. maltosaLEU2*	^22^

**Table 2:** Minimum inhibitory concentrations (MIC_80_) for shikonin (SK; μg/mL) against *Candida albicans*

***Candida albicans***			
	**Wild type**	***cta4Δ/Δ***	***yhb1Δ/Δ***
SK	8	4	4
